# On-surface lithium donor reaction enables decarbonated lithium garnets and compatible interfaces within cathodes

**DOI:** 10.1038/s41467-020-19417-1

**Published:** 2020-11-02

**Authors:** Ya-Nan Yang, Ying-Xiang Li, Yi-Qiu Li, Tao Zhang

**Affiliations:** 1 State Key Lab of High Performance Ceramics and Superfine Microstructure, Shanghai Institute of Ceramics, Chinese Academy of Sciences, 1295 Dingxi Road, Shanghai, 200050 P.R. China; 2 Center of Materials Science and Optoelectronics Engineering, University of Chinese Academy of Sciences, Beijing, 100049 P.R. China

**Keywords:** Batteries, Batteries, Batteries

## Abstract

Lithium garnets have been widely studied as promising electrolytes that could enable the next-generation all-solid-state lithium batteries. However, upon exposure to atmospheric moisture and carbon dioxide, insulating lithium carbonate forms on the surface and deteriorates the interfaces within electrodes. Here, we report a scalable solid sintering method, defined by lithium donor reaction that allows for complete decarbonation of Li_6.4_La_3_Zr_1.4_Ta_0.6_O_12_ (LLZTO) and yields an active LiCoO_2_ layer for each garnet particle. The obtained LiCoO_2_ coated garnets composite is stable against air without any Li_2_CO_3_. Once working in a solid-state lithium battery, the LiCoO_2_-LLZTO@LiCoO_2_ composite cathode maintains 81% of the initial capacity after 180 cycles at 0.1 C. Eliminating CO_2_ evolution above 4.0 V is confirmed experimentally after transforming Li_2_CO_3_ into LiCoO_2_. These results indicate that Li_2_CO_3_ is no longer an obstacle, but a trigger of the intimate solid-solid interface. This strategy has been extended to develop a series of LLZTO@active layer materials.

## Abstract (web summary)

Formation of insulating lithium carbonate on surface of lithium garnets hinders their application as solid electrolyte in lithium ion batteries. Here the authors explore a scalable sintering approach to utilize the undesired Li_2_CO_3_ and improve the active material-electrolyte interface within cathodes.

## Introduction

Solid-state batteries (SSBs) with a high-capacity lithium metal anode are considered as the ultimate alternative to liquid lithium-ion batteries^1^, which not only exhibit higher energy density but also fundamentally solve the safety problems of the liquid batteries due to the utilization of non-flammable solid-state electrolytes (SSEs). However, the solid–solid interfaces between SSEs and electrodes cause a large inherent impedance in the SSBs. At the same time, the SSEs are completely non-wetting compared with the liquid electrolyte so that the electrolyte cannot be immersed in the cathode to construct lithium-ion transport pathways, slowing the diffusion of lithium ions between particles inside the cathode. In order to address this issue, solid electrolyte powders are often added to the cathodes and the interface between solid electrolytes and active materials is designed to increase the ionic conductivity and decrease the polarization of electrode^2–5^. In the recent year, the SSBs employing a garnet-structured Li_7_La_3_Zr_2_O_12_ (LLZO) electrolyte have shown significant promise in practical applications because the LLZO electrolyte has high lithium-ion conductivity and is stable to lithium metal, but again, the ionic conductivity inside the cathode is low due to the use of the non-wetting LLZO solid electrolyte piece. Some efforts have been made to build lithium-ion transport channels inside cathode to preparing high performance composite cathodes in LLZO-based SSBs. For instance, Wakayama et al. reported a three-dimensional bicontinuous composite cathode which increased the surface area of the interface between the active materials and the LLZO particles^6^. Broek et al. embedded the electrode materials to the porous LLZO electrolyte, and it is beneficial to converting of lithium ions inside the electrode^7^. Besides, the interface properties of the composite cathode can also be improved by forming a coated structure in which active materials are coated with the LLZO particles^8^.

Unfortunately, it has been reported that LLZO is unstable in moist air and it is spontaneous to react with H_2_O and CO_2_ to generate a Li_2_CO_3_ layer on the surface^9^. The Li_2_CO_3_ layer is lithiophobicity and has an ultralow low lithium-ion conductivity so that it is one of the sources of the high interfacial impedance in SSEs^10,11^. So far, although it has been reported that Li_2_CO_3_ on the surface of LLZO can be removed by surface polishing^11^ or chemical reaction^12^, these approaches based on “eliminating” concept have just short-term effectiveness and in particular, only suitable for handling the large-sized surface of electrolyte piece. In contrast, a method for removing the Li_2_CO_3_ layer on the surface of the LLZO powder that has a larger surface area with more Li_2_CO_3_ has not been reported. This hinders the fast transport of lithium-ions inside cathode when adding LLZO powder to the cathode as an ion conductor or designing the internal interface inside the cathode. Therefore, reliable solutions to remove the Li_2_CO_3_ layer and to establish intimate physical contact between LLZO and active cathode materials are still needed.

Herein, we propose an “interface homogeneity” strategy to transform the Li_2_CO_3_ into LiCoO_2_ active material on the surface of Li_6.4_La_3_Zr_1.4_Ta_0.6_O_12_ (LLZTO) by an on-surface lithium-donor reaction. Significantly, the LLZTO coated with LiCoO_2_ (LLZTO@LCO) was obtained by the reaction of the Li_2_CO_3_ layer on the surface of LLZTO with Co_3_O_4_. The transformation from Li_2_CO_3_ into Li_2_CoO_2_ is complete and not reversible, indicating that the Li_2_CO_3_ layer can be fully removed. The formed LiCoO_2_ layer ensures direct contact between the solid electrolyte particles and the homogeneous LiCoO_2_ cathode material, circumventing the conventional heterogeneous solid–solid interface problem inside composite cathodes. In this work, the proposed LLZTO@LCO materials were successfully synthesized and characterized. For comparison, the LLZTO coated with naturally formed Li_2_CO_3_ (LLZTO@Li_2_CO_3_) and the LLZTO@LCO were used as an ionic conductor to prepare composite cathodes with LiCoO_2_ active materials, separately. And then LLZTO-based SSBs were assembled. We found that the battery with an LLZTO@LCO-containing LiCoO_2_ composite cathode exhibited a high Coulombic efficiency (CE) and improved cycling performance.

## Results

### Characterization of LLZTO and LLZTO@LCO

**Fig. 1:**
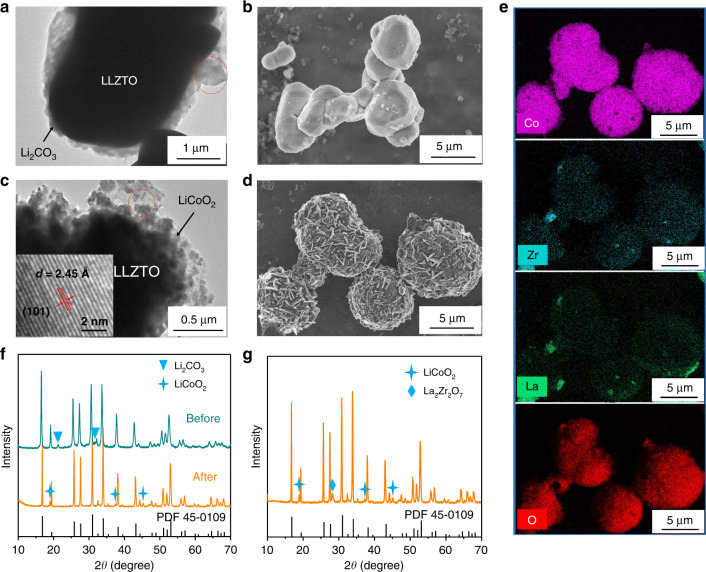
Characterization of LLZTO@Li_2_CO_3_ and LLZTO@LCO. **a**, **b** TEM image and SEM image of LLZTO powder exposed to air for four weeks. **c**, **d** TEM image and SEM image of LLZTO@LCO. **e** EDX mapping analysis of LLZTO@LCO corresponding to (**d**). **f** XRD patterns of LLZTO@Li_2_CO_3_ and LLZTO@ LCO. **g** XRD pattern of materials after first sintering containing impurity.

The LLZTO@LCO materials were prepared by a two-step solid sintering process. Figure 1a shows the transmission electron microscopy (TEM) image of LLZTO after air exposure for 4 weeks. It can be seen that a 0.1 μm thick layer formed on the surface of LLZTO. The coating layer can be indexed to the monoclinic Li_2_CO_3_ by subjecting selected area electron diffraction of the encircled region (Fig. S1). Figure 1b shows the scanning electron microscopy (SEM) image of LLZTO, the particle size of LLZTO is about 4 μm and has a smooth surface as well as irregular shape. After the reaction, instead of Li_2_CO_3_ layer, LiCoO_2_ is evenly distributed on the surface of LLZTO to form a coating with a thickness in the range of 0.3–0.5 μm, as shown in Fig. 1c. The inset in Fig. 1c shows the high resolution TEM (HRTEM) image of the encircled region. The lattice spacing of 0.245 nm agrees with the (101) facets of crystallized LiCoO_2_, and the widely exposed (101) facets (Fig. S2) exhibit higher ionic conductivity and electrochemical activity^13^. Moreover, as Fig. 1d shows, the LLZTO@LCO exhibits a spherical structure, which can increase the stacking density of composite cathode. The LiCoO_2_ exhibits a nanoplate-like, which is beneficial to the rate performance of the battery^14^. Figure 1e shows the energy-dispersive X-ray mapping analysis results. The Co, O elements are uniformly distributed on the surface of particles and the La, Zr elements can also be detected in that region, corresponding to the LLZTO@LCO structure. The X-ray diffraction (XRD) patterns, as shown in Fig. 1f, are in agreement with the TEM and SEM results. Compared with Li_5_La_3_Nb_2_O_12_ PDF card (45-0109), the existence of Li_2_CO_3_ on the surface of LLZTO was confirmed before reaction. Apparently, the ultimate materials only contain LLZTO and LiCoO_2_ after the reaction, and the LiCoO_2_ coating has R-3m symmetry as the traditional LiCoO_2_ active materials^15^. Above results confirm that the transformation of Li_2_CO_3_ to LiCoO_2_ is well achieved, but we find that an impurity is also formed during the first sintering. Figure 1g shows the XRD pattern of the lithium-donor reaction products after the first sintering. It should be noted that the impurity is La_2_Zr_2_O_7_. This is due to the volatilization of lithium from LLZTO during sintering^16^. Besides, after first sintering, the substitution of Li_2_CO_3_ by LiCoO_2_ indicates that the Li_2_CO_3_ coating has been fully reacted with Co_3_O_4_ to generate LiCoO_2_, but the LiCoO_2_ exhibits a block-like rather than a nanoplate-like (Fig. S3).

### Reaction mechanism of transforming LLZTO@Li_2_CO_3_ to LLZTO@LCO

**Fig. 2:**
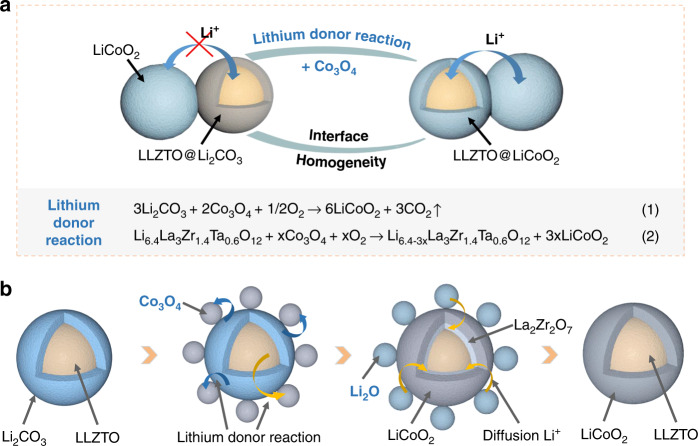
Process of transforming LLZTO@Li_2_CO_3_ into LLZTO@LCO. **a** Schematic illustration of the lithium-donor reaction to achieve interface homogeneity. **b** Schematic illustration of the two-step solid state reaction process of transforming LLZTO@Li_2_CO_3_ into LLZTO@LCO.

As shown in Fig. 2a, transforming LLZTO@Li_2_CO_3_ to LLZTO@LCO was achieved by the on-surface lithium-donor reaction. By sintering different rations of Co_3_O_4_ and LLZTO@Li_2_CO_3_, we found that there is no Co_3_O_4_ left after the excessive Co_3_O_4_ reacts with a small amount of Li_2_CO_3_ from the surface of LLZTO (Fig. S4). This is because, in addition to the reaction of Li_2_CO_3_ with Co_3_O_4_, there is also a reaction between lithium volatiles from LLZTO and the remaining Co_3_O_4_ to generate LiCoO_2_. Li_2_CO_3_ and LLZTO as the lithium donors together provide the lithium sources for the transformation reaction to form the complete LiCoO_2_ coating on the surface of LLZTO. To confirm the above process, we designed two experiments. First, Co_3_O_4_ with Li_2_CO_3_ materials were sintered under the first sintering condition and the result indicates that Co_3_O_4_ can react with Li_2_CO_3_ to generate LiCoO_2_ under this condition (Fig. S5). Second, we designed an experiment of sintering Li_2_CO_3_-free LLZTO and Co_3_O_4_ under the same condition. The La_2_Zr_2_O_7_ and LiCoO_2_ were still found in the sintered products, indicating that lithium volatilization exists in LLZTO during the sintering process, and the volatilized lithium can react with Co_3_O_4_ to form LiCoO_2_ (Fig. S6).

Based on the experimental results, Fig. 2b summarizes the process of the transformation. Initially, LLZTO exposed to air will form a Li_2_CO_3_ layer on the surface. Then, Co_3_O_4_ and LLZTO are fully mixed and sintered at 600 °C in air for 4 h. The Li_2_CO_3_ layer and Co_3_O_4_ undergo lithium-donor reaction to generate LiCoO_2_ on the surface of LLZTO. Meanwhile, the lithium source inside LLZTO also reacts with Co_3_O_4_ to generate LiCoO_2_ layer, but Li_6.4-3x_La_3_Zr_1.4_Ta_0.6_O_12_ lithium-deficient phase is formed due to the loss of lithium, which contains many lithium defects and leads to the formation of La_2_Zr_2_O_7_^17^. After that, in order to supply La_2_Zr_2_O_7_ with lithium-ion and let it return to the original LLZTO structure, Li_2_O salt is added and sintered again at 600 °C for 5 h in air. Excitingly, the lithiumization of La_2_Zr_2_O_7_ to LLZTO is realized. This process is the same as the preparation of LLZTO materials^18^. Finally, the pure LLZTO@LCO is obtained by washing and centrifuging. Different sintering temperatures (600, 700, 800, 900 °C) were attempted and we found that LiCoO_2_ can be synthesized at all of the above temperatures. However, when the temperature is higher than 700 °C, the diffusion of elements occurs (Fig. S7), which is consistent with previously reported^19,20^. In addition, we also tried the one-step sintering of LLZTO@Li_2_CO_3_, Co_3_O_4_, and Li_2_O, but the structure of LLZTO coated with LiCoO_2_ could not be formed and Li_2_CO_3_ still existed in store (Fig. S8).

### Stability and electrochemical activity of LLZTO@LCO

**Fig. 3:**
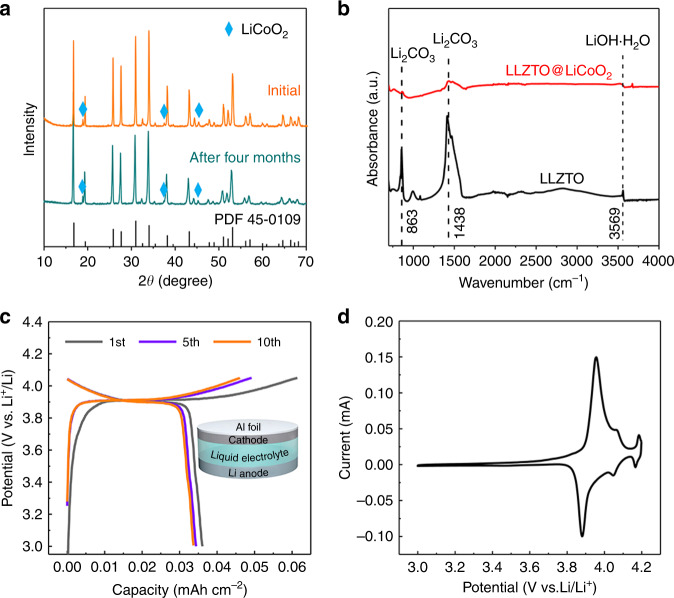
Stability and activity test of LLZTO@LCO. **a** XRD patterns of LLZTO@LCO before and after exposure to air for four months. **b** FTIR spectra of the LLZTO and LLZTO@LCO samples after exposure to air for four months. **c**, **d** Charge/Discharge curves and cyclic voltammetry profile of the liquid cells. The inset in Fig. 3c shows the structural illustration of the cell, in which the cathode was prepared by mixing LLZTO@LCO, PVDF and KB.

LLZTO@LCO was then subjected to air-stability and activity measurements. To clarify its stability, the LLZTO@LCO was exposed to air for 4 months. The XRD results are shown in Fig. 3a. It is obvious that Li_2_CO_3_ was not formed after exposure to air for a long time. This means that the LiCoO_2_ coating can restrain the formation of Li_2_CO_3_ layer and improve the stability of LLZTO significantly. The Fourier transform infrared (FTIR) spectra of the LLZTO and LLZTO@LCO samples exposed to air for 4 months also verified this result (Fig. 3b). It can be seen that the strong peaks of 1438 and 863 cm^−1^ were formed in LLZTO, which corresponds to the Li_2_CO_3_ FTIR spectrum^9^. In addition, the weak peak of 3569 cm^−1^ agrees with the LiOH H_2_O FTIR spectrum, which is due to the reaction between water and LLZTO^9,10^, but it does not affect the formation of LiCoO_2_ layer (Fig. S9). Conversely, Li_2_CO_3_ and LiOH H_2_O were not formed in LLZTO@LCO. To investigate lithium-intercalated activity of the LLZTO@LCO, it is used as the active material to prepare a cathode with a binder and conductive carbon, without the addition of any other active materials. A Li/LiClO_4_-EC-DEC/LLZTO@LCO liquid cell was then assembled (the inset in Fig. 3c). It should be noted that the liquid electrolyte is used here to describe the lithium-intercalated behavior of the LLZTO@LCO in more detail, so that it can be accurately compared with the characteristic redox peaks and charging/discharging voltage platforms (~3.9 V) of commercial LiCoO_2_. As shown in Fig. 3c, charging and discharging voltage platforms are confirmed at about 3.9 V, which is corresponding to that of the commercial LiCoO_2_. Figure 3d shows the cyclic voltammetry (CV) of the battery. There are strong redox peaks at about 3.87 and 3.95 V, indicating that the LiCoO_2_ coating in LLZTO@LCO exhibits activity, improving the transport of ions on the LLZTO surface. Two weak peaks appear at 4.05 and 4.2 V, where LiCoO_2_ lattice changes from hexagonal to monoclinic^21^.

### Characterization and ionic transport mechanism of the LCO-LLZTO@LCO composite cathode

**Fig. 4:**
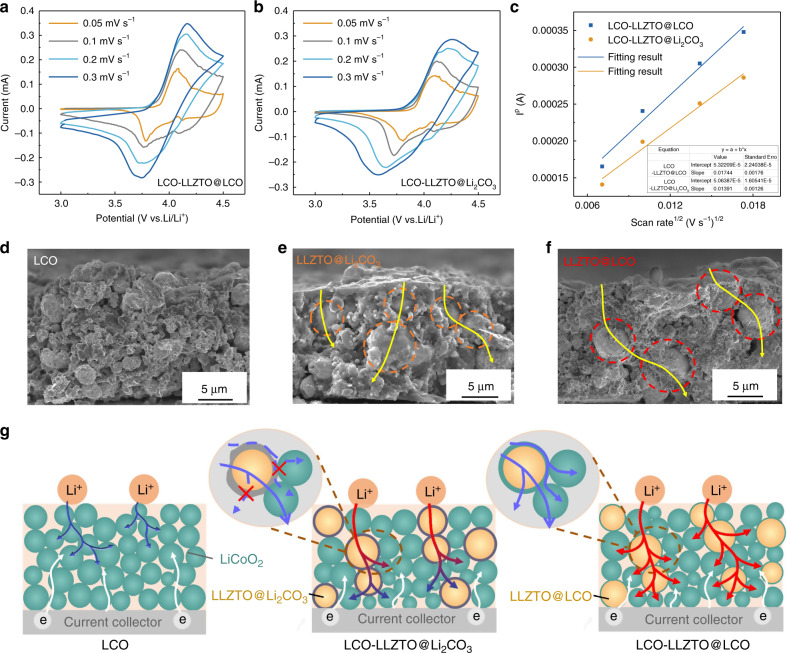
Characterization and ionic transport mechanism of the cathodes. CV profiles of **a** LCO-LLZTO@LCO and **b** LCO-LLZTO@Li_2_CO_3_ cathodes at different scan rates. **c** Peak current as a function of the square root of the scan rate of LCO-LLZTO@LCO and LCO-LLZTO@Li_2_CO_3_ cathodes. **d**–**f** Cross-sectional SEM images of the LiCoO_2_, LCO-LLZTO@Li_2_CO_3_ and LCO-LLZTO@LCO cathodes, separately. **g** Schematic illustration of the ionic transport mechanism inside cathodes.

In order to investigate the effect of LLZTO@LCO on the lithium-ion transfer kinetics of the cathode, the lithium-ion apparent diffusion coefficient was tested by performing CV measurements. Figure 4a, b shows the CV profiles of the LCO-LLZTO@LCO and LCO-LLZTO@Li_2_CO_3_ cathodes at different scan rates. The lithium-ion apparent diffusion coefficient can be calculated according to the Randles–Sevcik equation^22^

1 $$I_p = 2.68 \times 10^5n^{3/2}A\,C\,D^{1/2}v^{1/2},$$

where *I*_*p*_ is the peak current (A); *n* is the charge-transfer number of the redox reaction; *A* is the area of the cathode plate (cm^2^); *C* is the lithium-ion concentration in LiCoO_2_ cathode (0.051 mol cm^−3^); *D* is the lithium-ion diffusion coefficient (cm^2^ s^−1^); *v* is the scan rate (V s^−1^). *I*_*p*_ is linearly related to *v*^1/2^ and the value of *I*_*p*_*/v*^1/2^ can be obtained from the linear fitting results as 0.01744 and 0.01391 for LCO-LLZTO@LCO and LCO-LLZTO@Li_2_CO_3_ cathodes (Fig. 4c). The lithium-ion apparent diffusion coefficient could be calculated to be 2.04 × 10^−13^ cm^2^ s^−1^ for LCO-LLZTO@LCO cathode and 1.28 × 10^−13^ cm^2^ s^−1^ for LCO-LLZTO@Li_2_CO_3_ cathode. Significantly, after Li_2_CO_3_ is converted to LiCoO_2_, the lithium-ion diffusion coefficient of the cathode is increased by about 59%. This is because the activated LLZTO@LCO promotes ionic transport between particles, decreasing the resistance inside cathode. (Fig. S10).

To explore the mechanism by which LLZTO@LCO particles enhance the transport of lithium ions inside cathode, the cross-sectional micromorphology of the cathodes were observed. As shown in Fig. 4d, before adding LLZTO to the LiCoO_2_ cathode, the LiCoO_2_ nanoparticles are distributed on the cathode layer with a thickness of 15 μm, constructing a lithium-ion transport network. After LLZTO@Li_2_CO_3_ and LLZTO@LCO are introduced (Fig. 4e, f), they are distributed throughout the cathode and are in close contact with LiCoO_2_ particles around, providing composite channels for lithium-ion transport. Significantly, LLZTO@Li_2_CO_3_ and LLZTO@LCO particles with a large particle size cross the cathode layer, which can construct rapid large-span channels for the transport of the lithium ions to the interior of the cathode. However, the Li_2_CO_3_ layer on the surface of LLZTO with low conductivity will increase the interface impedance between the active material and the ion conductor particles. Conversely, LLZTO@LCO particles not only have an active surface in close contact with the active materials (Fig. S11), but also have a tight and low-impedance interface at the junction of LLZTO core and LiCoO_2_ shell (Figs. S12 and 13), promoting the transport of lithium ions inside the cathode.

Based on the above results, it can be concluded that the optimization of the transport channels for lithium ions by LLZTO@LCO may be the reason for the improved ionic conductivity, thus a possible mechanism is provided in Fig. 4g. In the LiCoO_2_ cathode without an ionic conductor, lithium ions diffuse into cathode through the ionic channels constructed by the active material LiCoO_2_ with low ionic conductivity, which can reduce the diffusion rate and diffusion depth of lithium ions, causing a large voltage polarization and limiting electrode reaction to occur in the shallow layer of the cathode. But, after LLZTO@Li_2_CO_3_ is added, large-span transport channels for lithium ions are formed around LLZTO@Li_2_CO_3_ particles, which can quickly transport lithium ions deeper into the LCO-LLZTO@ Li_2_CO_3_ composite cathode. But, the presence of Li_2_CO_3_ on the surface of LLZTO is like sludge in the channels. Lithium ions can only enter and exit LLZTO particles from the thin layer of Li_2_CO_3_ and can only be transported to LiCoO_2_ particles through the LLZTO bulk phase (Fig. S14), limiting the diffusion direction of lithium ions to the periphery, short of a crisscross lithium-ion transport network. Transforming Li_2_CO_3_ layer into active LiCoO_2_ layer is like dredging the channels. Lithium ions can be freely transported not only in the bulk phase but also the surface of LLZTO@LCO, allowing the rapid lithium-ion transport paths to branch in any direction (Fig. S14), which realizes uniform diffusion of lithium ions on the shallow and deep layer of the LCO-LLZTO@LCO composite cathode. The rapid lithium-ion transport channel can be compared to an irrigation canal, in which the main channel is constructed along the high ionic conductivity area where more LLZTO@LCO particles are distributed, and the main channel branches to the surroundings to deliver lithium ions to various locations of the cathode, which greatly improves the transport efficiency of lithium ions. This transport mechanism allows lithium ions to be quickly and evenly distributed throughout the cathode, so the LCO-LLZTO@LCO composite cathode exhibits higher ionic conductivity.

### LLZTO-based SSBs with LCO-LLZTO@LCO composite cathode

**Fig. 5:**
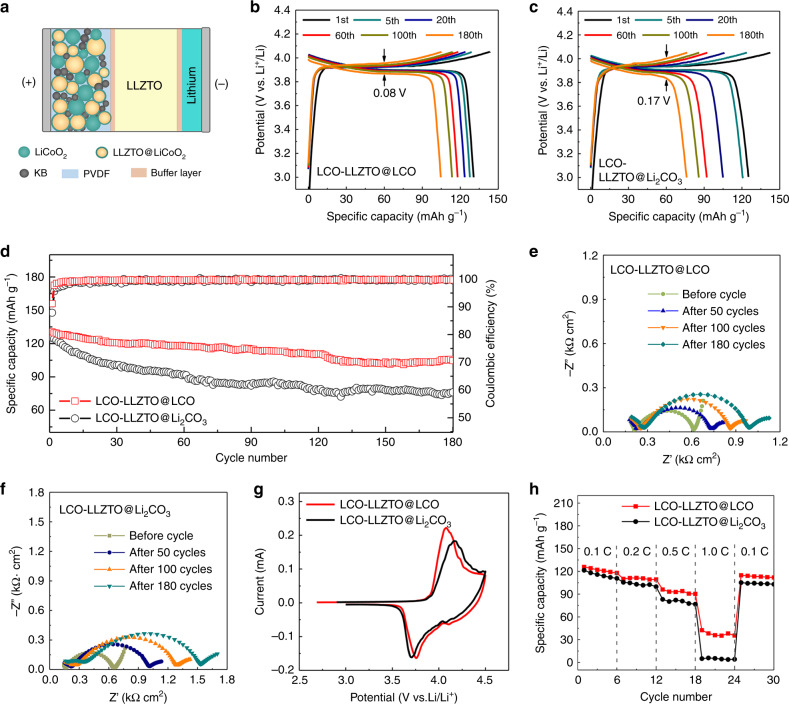
Electrochemical performance of the LLZTO-based SSBs. **a** Schematic illustration of the LLZTO-based SSB. The buffer layer was formed by dissolving 10 wt% of lithium trifluoromethanesulfonyl (LiTFSI) in succinonitrile (SN), and polyacrylonitrile (PAN) was added to enhance film-forming property. **b**, **c** Discharge/charge curves of the SSBs with an LCO-LLZTO@LCO and LCO-LLZTO@Li_2_CO_3_ cathode, separately. **d** Cycling performance of the SSBs with an LCO-LLZTO@LCO and LCO-LLZTO@Li_2_CO_3_ cathode, separately. **e**, **f** EIS of the SSBs with a LCO-LLZTO@LCO and LCO-LLZTO@Li_2_CO_3_ cathode, separately. **g** CVs of the SSBs with an LCO-LLZTO@LCO and LCO-LLZTO@Li_2_CO_3_ cathode, separately. **h** Rate capability of the SSBs with an LCO-LLZTO@LCO and LCO-LLZTO@Li_2_CO_3_ cathode, separately. All tests were performed at room temperature.

The electrochemical properties of the LLZTO@LCO and LLZTO@Li_2_CO_3_ were also compared in SSBs consisting of a lithium anode and LLZTO solid electrolyte pellet. The commercial LiCoO_2_ was used as the active material, and LLZTO@Li_2_CO_3_ and LLZTO@LiCoO_2_ were used as ionic conductors to prepare composite cathodes, separately. Then, the prepared LLZTO@Li_2_CO_3_-containing LiCoO_2_ (LCO-LLZTO@Li_2_CO_3_) cathodes and LLZTO@LCO-containing LiCoO_2_ (LCO-LLZTO@LCO) cathodes were assembled into coin-type cells separately (Fig. 5a). Meanwhile, a thin buffer layer that is solid at room temperature was used to reduce the interface impedance between the electrolyte piece and the electrode plates (Fig. S15). All the cells were cycled at room temperature as well as 0.1 C (1 C = 140 mA g^−1^). The cycling performance of the battery with an LCO-LLZTO@LCO cathode is shown in Fig. 5b. The discharge capacity of the first cycle reached 131 mA h g^−1^ and the discharge capacity can be retained at 81% after 180 cycles with a voltage polarization of 0.08 V. Moreover, after 180 cycles the structure of LLZTO@LCO particles remained stable (Fig. S16). In contrast, the capacity retention of the battery with an LCO-LLZTO@Li_2_CO_3_ cathode reached only 60% after 180 cycles (Fig. 5c), but better than that of the battery with a pure LiCoO_2_ cathode without an LLZTO@LCO or LLZTO@Li_2_CO_3_ ionic conductor (Fig. S17). Furthermore, the CE of the battery with an LCO-LLZTO@LCO cathode reaches 91.1% at first cycle and stable at above 99% after the first five cycles, but the battery with an LCO-LLZTO@Li_2_CO_3_ cathode exhibited lower CE of 87.8% at first cycle (Fig. 5d). The increase in CE is owing to the reduction of side reactions by removing the Li_2_CO_3_ inside the cathode. Figure 5e, f shows the electrochemical impedance spectroscopy of the batteries. It can be seen that the impedance plot includes an incomplete semicircle in the high frequency region, a semicircle in the middle frequency region and a tail in low frequency region, in which the semicircle in the middle frequency region corresponds to the overall interface resistance (*R*_int_) inside the battery. The interface *R*_int_ in the battery with an LCO-LLZTO@LCO cathode is 600 Ω cm^2^ after 180 cycles, lower than 988 Ω cm^2^ in the battery with an LCO-LLZTO@Li_2_CO_3_ cathode (Fig. S18). The smaller interface resistance is mainly due to the optimized ion transfer channels of the LLZTO@LCO particles. In addition, the CVs measured on the composite cathodes (Fig. 5g) show that LCO-LLZTO@LCO cathode has a lower polarization. This can be explained by the fact that the transformation of insulating Li_2_CO_3_ to active LiCoO_2_ with high ionic conductivity achieves interface homogeneity inside cathode and can speed up the transport of lithium-ion between the particles. The change of LiCoO_2_ lattice from hexagonal to monoclinic is also observed at about 4.05 and 4.2 V by testing d*Q* d*V*^−1^ of the SSB with an LCO-LLZTO@LCO cathode (Fig. S19), but that is not obviously shown in the CV curve in Fig. 5g. This is because the SSB has a higher impedance than the liquid battery, leading to a larger polarization, which results in a shift and widen in the main peak of the LiCoO_2_ so that the weak peaks at 4.05 and 4.2 V are covered. Figure 5h shows the rate performance of the batteries. The discharge capacity of the battery with an LCO-LLZTO@LCO cathode still reached 116 mA h g^−1^ at 0.2 C and 100 mA h g^−1^ at 0.5 C, but the discharge capacity of the battery with an LCO-LLZTO@Li_2_CO_3_ cathode only reached 105 and 80 mA h g^−1^ at 0.2 and 0.5 C, which corresponds to higher ionic conduction of the LCO-LLZTO@LCO cathode. Significantly, instead of using low-voltage active materials which were mostly used in the LLZO-based SSBs in previous reports, the high-voltage LiCoO_2_ active materials with LLZTO@LCO ionic conductor are used to prepare composite cathodes to assemble solid cells in this work, and show improved cycleability and rate performance (Fig. S20).

## Discussion

Purity of electrolytes has guided the history of commercial batteries. For instance, the successful development of high-purity LiPF_6_ in 1994, coupled with 99.9% pure ethylene carbonate, offered a leap forward in cycling ability of commercial lithium-ion batteries. The ubiquitous Li_2_CO_3_ can be considered as an impurity of LLZO particles, leading to inferior purity (<95%), which is far away from the practical needs. Hence, when using in LiCoO_2_ cathodes, the purity of the LLZO electrolyte is equivalent to 100% owing to the substitution of insulating Li_2_CO_3_ impurity for active LiCoO_2_, and the latter provides a homogeneous contact with the LiCoO_2_ active material inside composite cathodes. This is an apparent advantage of the LLZTO@LCO from the view point of electrolyte purity.

**Fig. 6:**
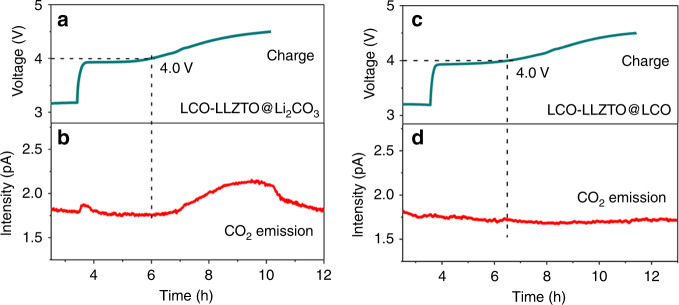
DEMS analysis of LLZTO@Li_2_CO_3_ and LLZTO@LCO in composite cathodes. **a**, **b** Charge curves (top) and corresponding CO_2_ emission (bottom) of LCO-LLZTO@Li_2_CO_3_ and LCO-LLZTO@LCO cathode, separately.

To clarify the electrochemical difference of LLZTO@Li_2_CO_3_ and LLZTO@LCO inside the composite cathode, the LCO-LLZTO@Li_2_CO_3_ and LCO-LLZTO@LCO composite cathodes were analyzed by the differential electrochemical mass spectrometry analysis (DEMS) separately. The assembled liquid batteries were used to detect the release of CO_2_. Figure 6a shows the charge curve (top) and corresponding CO_2_ emission (bottom) for the LCO-LLZTO@Li_2_CO_3_ composite cathode. Notably, the intensity of CO_2_ began to increase when charged to about 4.0 V (vs. Li/Li^+^), which is considered to be due to the decomposition of Li_2_CO_3_ in LLZTO@Li_2_CO_3_, consistent with previous observations that Li_2_CO_3_ was decomposed above 4.0 V^23,24^. It should be noted that this is the first demonstration of the electrochemical decomposition of Li_2_CO_3_ formed on the surface of LLZTO by experiments. In stark contrast, benefiting from the transformation of Li_2_CO_3_ into LiCoO_2_, CO_2_ was not released in the homogeneous LCO-LLZTO@LCO composite cathode (Fig. 6b), exhibiting much higher electrochemical stability, which explains the high initial CE of the battery with an LCO-LLZTO@LCO composite cathode.

**Fig. 7:**
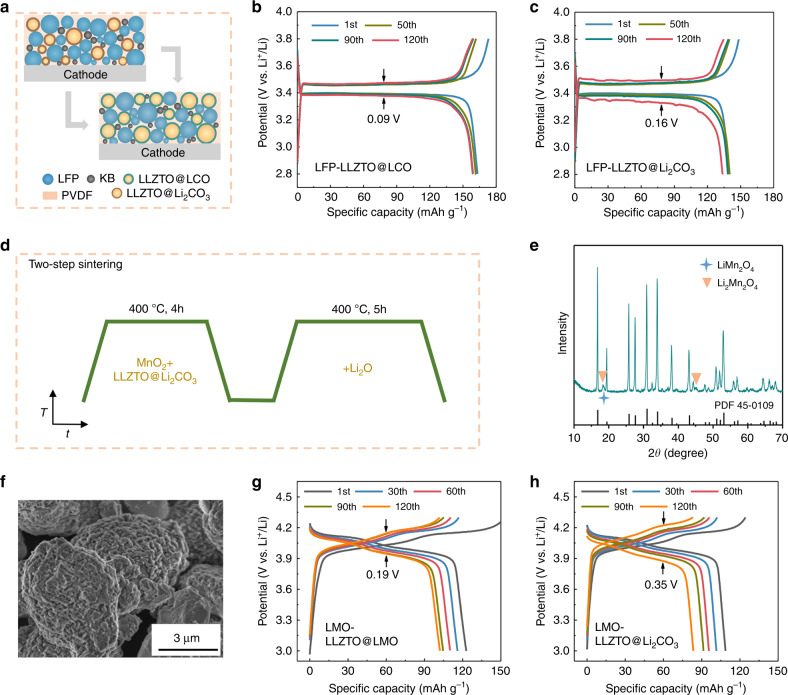
Extensive applicability of LLZTO@active-material and the two-step solid state reaction. **a** Schematic illustration of the LFP-LLZTO@Li_2_CO_3_ and LFP-LLZTO@LCO composite cathodes. **b**, **c** Discharge/charge curves of the SSBs with an LFP-LLZTO@LCO and LFP-LLZTO@Li_2_CO_3_ composite cathode, separately. **d** Schematic illustration of the two-step solid state reaction. **e**, **f** SEM image and XRD pattern of the ultimate materials after converting Li_2_CO_3_ into LMO. **g**, **h** Discharge/charge curves of the SSBs with an LMO-LLZTO@LMO and LMO-LLZTO@Li_2_CO_3_ composite cathode, separately.

We further evaluated the activity and extensive applicability of LLZTO@LCO by adding it to LiFePO_4_ cathodes with a low ionic diffusion rate. The prepared LLZTO@LCO-containing LiFePO_4_ (LFP-LLZTO@LCO) composite cathode and LLZTO@Li_2_CO_3_-containing LiFePO_4_ (LFP-LLZTO@Li_2_CO_3_) composite cathode (Fig. 7a) were assembled into SSBs using a lithium anode and a LLZTO solid electrolyte pellet, separately. As shown in Fig. 7b, the initial discharge capacity of the battery with an LFP-LLZTO@LCO cathode reached 163.2 mA h g^−1^, closing to the theoretical capacity of 170 mA g^−1^, and can be retained at 97% after 120 cycles with a low-voltage polarization of 0.09 V. In contrast, the battery with an LFP-LLZTO@Li_2_CO_3_ cathode exhibits an initial discharge capacity of 146.4 mA h g^−1^ and has a large voltage polarization of 0.16 V after 120 cycles (Fig. 7c). In addition, the SSB with an LFP-LLZTO@LCO cathode exhibits longer-term cycling performance than that previously reported (Fig. S21)^4,25–29^.

The two-step solid-state reaction process (Fig. 7d) was also successfully extended to prepare LLZTO@LiMn_2_O_4_. Figure 7e shows the XRD pattern of the converted materials. The ultimate materials contain LLZTO, LiMn_2_O_4_, and Li_2_Mn_2_O_4_, in which the Li_2_Mn_2_O_4_ is a discharged state of LiMn_2_O_4_ due to the presence of excess lithium salt during sintering, causing the insertion of lithium ions into LiMn_2_O_4_. Figure 7f shows the SEM image of LLZTO@LiMn_2_O_4_/Li_2_Mn_2_O_4_ (LLZTO@LMO). LiMn_2_O_4_ and Li_2_Mn_2_O_4_ with a nanoparticle-like morphology are evenly distributed on the surface of LLZTO to form a coating. The electrochemical properties of the LLZTO@LMO-containing LiMn_2_O_4_ (LMO-LLZTO@LMO) and LLZTO@Li_2_CO_3_-containing LiMn_2_O_4_ (LMO-LLZTO@Li_2_CO_3_) composite cathodes were compared in SSBs with the lithium anode and LLZTO electrolyte pellet. The initial discharge capacity of the battery with an LMO-LLZTO@LMO cathode reached 122.8 mA h g^−1^, which is higher than that of another battery (108.7 mA h g^−1^). In addition, compared with the battery with an LMO-LLZTO@Li_2_CO_3_ cathode, the battery with an LMO-LLZTO@LMO cathode exhibits improved cycling stability (Fig. S22) and a lower voltage polarization of 0.19 V after 120 cycles at 0.1 C (1 C = 148 mA g^−1^), while the initial CE is lower due to the presence of discharged Li_2_Mn_2_O_4_ (Fig. 7g, h).

In conclusion, by transforming the ubiquitous insulating Li_2_CO_3_ layer on the surface of LLZTO solid electrolytes into an active LiCoO_2_ layer, pure LLZTO@LCO particles are successfully synthesized with an on-surface lithium-donor reaction. The R-3m symmetry LiCoO_2_ is mainly generated by the lithium donor reaction of the Li_2_CO_3_ layer and Co_3_O_4_ in the first sintering. At the same time, it is found that lithium volatiles from LLZTO also reacts with Co_3_O_4_, resulting in part of LiCoO_2_, accompanying by La_2_Zr_2_O_7_ impurity. At the heart of our technology is offsetting the formidable impurity La_2_Zr_2_O_7_ by precisely supplementing extra lithium sources, thus restoring it to the pristine LLZTO structure in the second sintering step. The LLZTO@LCO particles are exposed to air for 4 months without Li_2_CO_3_ formation, indicating excellent store stability and demonstrating a radical solution of the Li_2_CO_3_ issue. We found that the decomposition of Li_2_CO_3_ formed on the surface of LLZTO occurs at voltages above 4.0 V, which is one of the reasons for the low initial Coulomb efficiency. Meanwhile, the converted LiCoO_2_ layer of the LLZTO@LCO particles exhibits the same lithium-intercalated electrochemical activity with commercial LiCoO_2_, which enables it to interact favorably with the LiCoO_2_ active material in the solid LCO-LLZTO@LCO composite cathode. As a consequence of the interface homogeneity inside cathode, the solid-state lithium metal battery with the LLZTO@LCO and LiCoO_2_ composite cathode shows 81% capacity retention after 180 cycles at 0.1 C, room temperature, superior to that with the LLZTO@Li_2_CO_3_ and LiCoO_2_ one, representing the highest level among LiCoO_2_-based solid batteries. Our results indicate that although the formation of Li_2_CO_3_ on LLZO is inevitable, it would no longer hinder. Lithium-ion transfer at the solid electrolyte/cathode interface, but provide a chance to be transformed into active materials, thus achieving an in-situ intimate contact of ion conductor and active materials inside the cathode. In addition, the solid sintering reaction, which is the most common mass production method for ceramics-type electrolytes and cathode materials, has also been successfully applied to the in-situ transformation of Li_2_CO_3_ to LiMn_2_O_4_. It is hopeful to develop a series of LLZO@LiFePO_4_, LLZO@layered Ni-Co-Mn or Ni-Co-Al, etc. to precisely match active materials inside the composite cathodes for solid-state lithium metal batteries.

## Methods

### LLZTO@LiCoO_2_ materials

LLZTO@LiCoO_2_ materials were prepared by a two-step solid-state reaction process. LLZTO powders and Co_3_O_4_ (Aladdin, 99.99%) were mixed in a mass ratio of 20:3 at an agate mortar for 10 min and sintered at 600 °C for 4 h. Then Li_2_O were added to the precursors in a mass ratio of 3:10, and heated to 600 °C and dwelled on for 5 h. The obtained materials were finally washed with ethyl alcohol and centrifuged giving rise to LLZTO@LiCoO_2_. The LLZTO powders and pellets was prepared by a method previously reported^30,31^.

### Composite cathodes

The LCO-LLZTO@LCO and LCO-LLZTO@Li_2_CO_3_ composite cathodes were prepared in the air. LLZTO@LiCoO_2_ and LLZTO@Li_2_CO_3_ solid electrolytes were, respectively, mixed with LiCoO_2_ active materials, PVDF, KB in a mass ratio of 3:5:1:1 in N-methylpyrrolidone (NMP) solvent. After stirring for 12 h, the slurry was scraped on the carbon-containing aluminum foil, and heated at 60 °C in atmospheric pressure for 2 h, then dried at 80 °C in vacuum for 24 h to obtain cathode foil and cut it into discs of 12 mm in diameter. A total of 80 wt% of commercial LiCoO_2_, 10 wt% of PVDF, and 10 wt% of KB were mixed to prepare pure LiCoO_2_ cathodes. The LFP-LLZTO@LCO and LFP- LLZTO@Li_2_CO_3_ composite cathodes were prepared by the same method as above.

### Assembly of liquid cells

CR2032-type liquid coin cells were assembled in an argon-filled glovebox to detect the air-stability and activity of LLZTO@LCO. The cathodes were prepared by mixing LLZTO@LiCoO_2_ active materials, PVDF and KB in a mass ratio of 8:1:1 in NMP solvent. After stirring for 12 h, the slurry was scraped on the carbon-containing aluminum foil, and heated at 60 °C in atmospheric pressure for 2 h, then dried at 80 °C in vacuum for 24 h to obtain cathode plate and cut it into discs of 12 mm in diameter. Li foil with 12 mm in diameter was used as anode and dissolving 0.1 M LiClO_4_ in EC-DEC (1:1, v/v) was used as a liquid electrolyte.

### Assembly of solid-state cells

CR2032-type solid-state coin cells were assembled in an argon-filled glovebox. In all-solid-state cells, the LLZTO plates were used as SSEs and Li foils with 12 mm in diameter were used as anodes. In order to improve the interface between SSE and electrodes, a buffer layer that exhibits a film at room temperature was introduced, which was formed by dissolving 10 wt% of trifluoromethanesulfonyl in succinonitrile at 80 °C and adding polyacrylonitrile to enhance film-forming property. The gelatinous slurry was scraped on the electrode surface at 80 °C, and cooled down to room temperature to form a solid film.

### Electrochemical measurement

The charge/discharge tests of the cells were carried out using Land machines at room temperature. The specific capacity of the batteries with an LCO-LLZTO@LCO cathode was calculated based on the weight of the cathode active materials including both the LiCoO_2_ on the surface of LLZTO@LiCoO_2_ and the commercial LiCoO_2_. The proportion of Co element is 11.58 wt% in the LLZTO@LiCoO_2_, which is provided by the inductively coupled plasma spectrum test. The loading of cathodes is about 2 mg cm^−2^, corresponding to the active materials of around 1.12 mg cm^−2^.

### TEM, selected area electron diffractions, and high resolution transmission electron microscopy observation

The coating structure of LLZTO@Li_2_CO_3_ and LLZTO@LiCoO_2_ were observed using Field Emission JEM-2100F TEM. The diffraction fringes and lattice fringes of LLZTO@Li_2_CO_3_ and LLZTO@LiCoO_2_ were observed using selected area electron diffractions and high resolution transmission electron microscopy of field emission JEM-2100F TEM, separately.

### SEM observation and energy-dispersive X-ray spectroscopy analysis

The microstructures and X-ray (energy-dispersive X-ray spectroscopy) mapping of LLZTO and LLZTO@LiCoO_2_ were observed using SU-8220 field emission SEM.

### X-ray powder diffraction and FTIR analysis

The phases and crystalline structure of the materials before and after transformation were analyzed using X-ray powder diffraction with Cu Kα radiation. LLZTO and LLZTO@LiCoO_2_ were exposed to air for four months for FTIR measurements.

### Differential electrochemical mass spectrometry analysis

The LCO-LLZTO@LCO and LCO-LLZTO@Li_2_CO_3_ slurry were dripped on stainless steel with 12 mm in diameter, separately. Then, the dried composite cathodes were assembled into customized Swagelok cells. Dissolving 1 M LiPF_6_ in EC/DMC (1:1 v:v) was used as the liquid electrolyte and Li foil with 12 mm in diameter was used as the anode. The liquid cells were charged to 4.5 V at 0.25 C.

## Supplementary information

Supplementary Information

Peer Review File
