# Supplementary material for: On-surface lithium donor reaction enables decarbonated lithium garnets and compatible interfaces within cathodes
Source: Nat Commun. 2020 Nov 2;11:5519. doi: 10.1038/s41467-020-19417-1 (PMC7608658; doi:10.1038/s41467-020-19417-1)
Supplement: Supplementary file 1 — Supplementary Information [file 41467_2020_19417_MOESM1_ESM.pdf]

Supporting Information

On-surface Lithium Donor Reaction Enables Decarbonated Lithium Garnets and Compatible Interfaces within Cathodes

Ya-Nan Yang[1,2], Ying-Xiang Li[1,2], Yi-Qiu Li[1], & Tao Zhang[1,2]*

[1]State Key Lab of High Performance Ceramics and Superfine microstructure, Shanghai Institute of Ceramics, Chinese Academy of Sciences, 1295 Dingxi Road, Shanghai, 200050, P. R. China

[2]Center of Materials Science and Optoelectronics Engineering, University of Chinese Academy of Sciences, Beijing 100049, P. R. China

Table of Contents

Supplementary Figures 1

Supplementary Figure 1: SAED image of the Li_2CO_3 layer on the surface of LLZTO........ 1

Supplementary Figure 2: HRTEM image of the $LiCoO_2$ layer on the surface of LLZTO 2

Supplementary Figure 3: SEM images of the LLZTO@LCO 3

Supplementary Figure 4: XRD patterns of LLZTO@Li_2CO_3 and Co_3O_4 sintered at different ratios. 4

Supplementary Figure 5: XRD patterns of the Li_2CO_3 and Co_3O_4 sintered at 600 °C.......... 5

Supplementary Figure 6: Sintering result of Li_2CO_3–free LLZTO and Co_3O_4........ 6

Supplementary Figure 7: XRD patterns of the LLZTO@Li_2CO_3 and Co_3O_4 sintered at different temperatures. 7

Supplementary Figure 8: Comparison of one-step sintering and two-step sintering 8

Supplementary Figure 9: Characterization and transformation of $LiOH{\cdot}H_2O$ 9

Supplementary Figure 10: EIS analysis of the cathodes without KB. 10

Supplementary Figure 11: SEM image of the LLZTO@LCO particle in the LCO-LLZTO@LCO composite cathode. 11

Supplementary Figure 12: Characterization of the interface between LLZTO and $LiCoO_2$ in LLZO@LCO particle 12

Supplementary Figure 13: EIS analysis of the cold-pressed pieces 13

Supplementary Figure 14: Lithium-ion transport paths on the LLZTO@Li_2CO_3 and LLZTO@LCO particles. 14

Supplementary Figure 15: Characterization of the buffer layer 15

Supplementary Figure 16: Stability of the structure of LLZTO@LCO after charge/discharge 180 cycles 16

Supplementary Figure 17: Electrochemical performance of the SSB with a commercial $LiCoO_2$ cathode. 17

Supplementary Figure 18: Equivalent circuit analysis of EIS for the SSBs 18

Supplementary Figure 19: dQ dV^{-1} curve of the SSB 19

Supplementary Figure 20: Cycle life of various $LiCoO_2$-based SSBs 20

Supplementary Figure 21: Cycling performance of the SSB with an LFP-LLZTO@LCO cathode. 21

Supplementary Figure 22: Cycling performance of the SSBs with an LMO, LMO-LLZTO@LMO and LMO-LLZTO@Li_2CO_3 cathode. 22

Supplementary Tables 23

Supplementary Table 1: Analysis results of equivalent circuits for EIS 23
Supplementary Table 2: Comparison of the cycle performance of the LLZO-based SSBs employing $LiFePO_4$ cathodes. 23
Supplementary Table 3: Fitting results of Au/cold-pressed piece/Au. 24
Supplementary Table 4: Fitting results of Au/cold-pressed LLZTO@Li_2CO_3/Au. 24
Supplementary Discussions 25
Supplementary Discussion 1 25
Supplementary Discussion 2 26
Supplementary Discussion 3 27
Supplementary References 28

Supplementary Figures

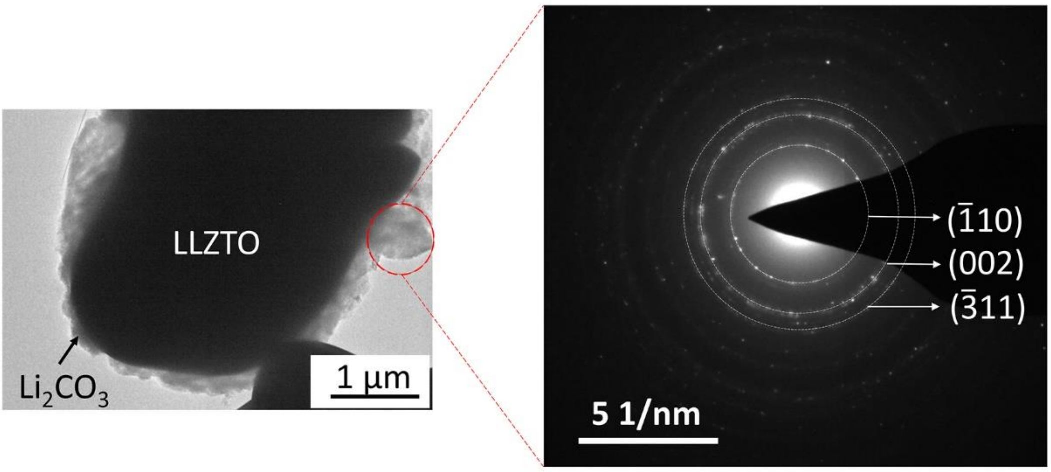

Supplementary Figure 1: SAED image of the Li_2CO_3 layer on the surface of LLZTO.

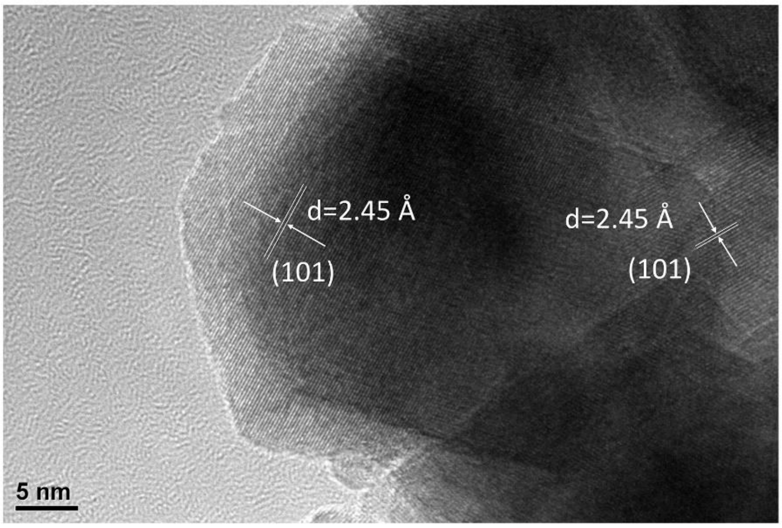

Supplementary Figure 2: HRTEM image of the $LiCoO_2$ layer on the surface of LLZTO. Most of the exposed facets of the stacked nanoplate-like $LiCoO_2$ are (101).

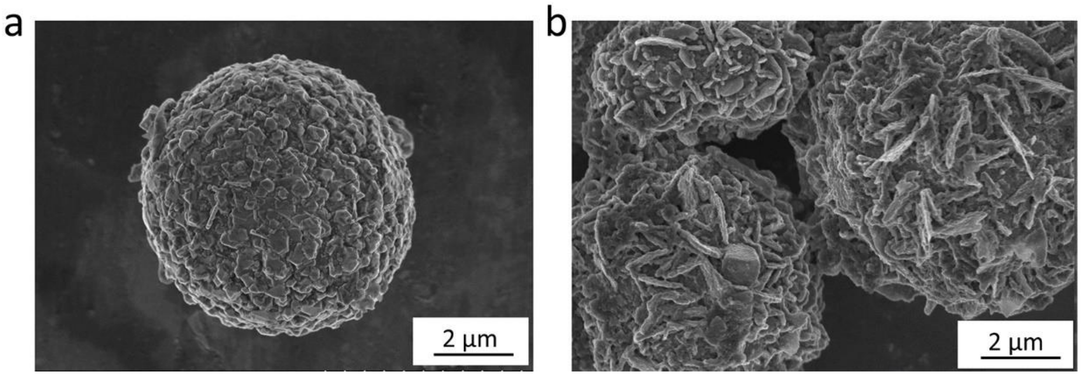

Supplementary Figure 3: SEM images of the LLZTO@LCO. a SEM image of the LLZTO@$LiCoO_2$ after first sintering. The $LiCoO_2$ coating has been formed, but exhibits a block-like. **b** SEM image of the LLZTO@$LiCoO_2$ after second sintering. The $LiCoO_2$ exhibits a nanoplate-like.

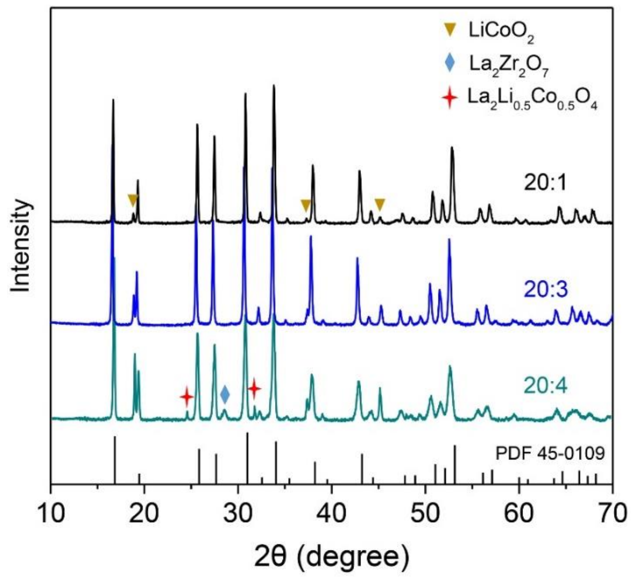

Supplementary Figure 4: XRD patterns of LLZTO@Li_2CO_3 and Co_3O_4 sintered at different ratios.

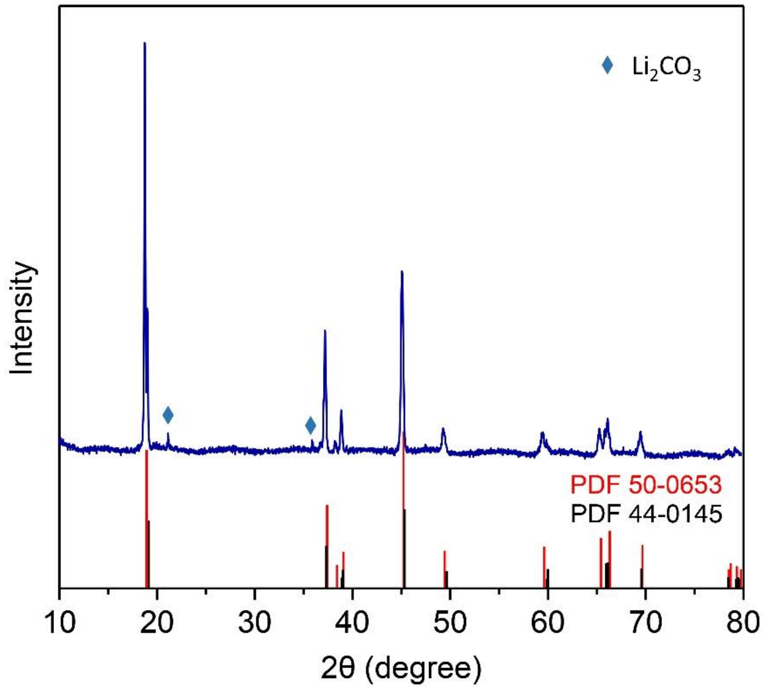

Supplementary Figure 5: XRD patterns of the Li_2CO_3 and Co_3O_4 sintered at 600 ^oC for 4 hours. Different $LiCoO_2$ materials (PDF 50-0653 and PDF 44-0145) have the same R-3m symmetry with slight differences in lattice parameters.

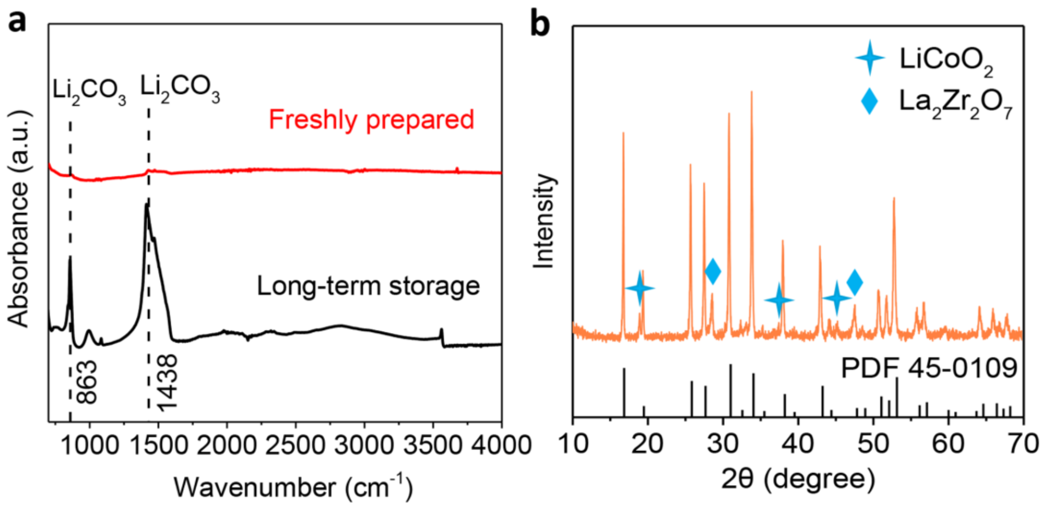

Supplementary Figure 6: Sintering result of Li_2CO_3–free LLZTO and Co_3O_4. a FTIR spectra of LLZTO prepared freshly or exposed to the atmosphere for a long time. There is almost no Li_2CO_3 on the surface of the freshly prepared LLZTO. **b** XRD pattern of Li_2CO_3-free LLZTO and Co_3O_4 sintered in an argon atmosphere at 600°C for 4h. The formation of $La_2Zr_2O_7$ indicates the presence of lithium volatilization in LLZTO. The generation of $LiCoO_2$ confirms that Co_3O_4 reacts with evaporated Li and generates $LiCoO_2$.

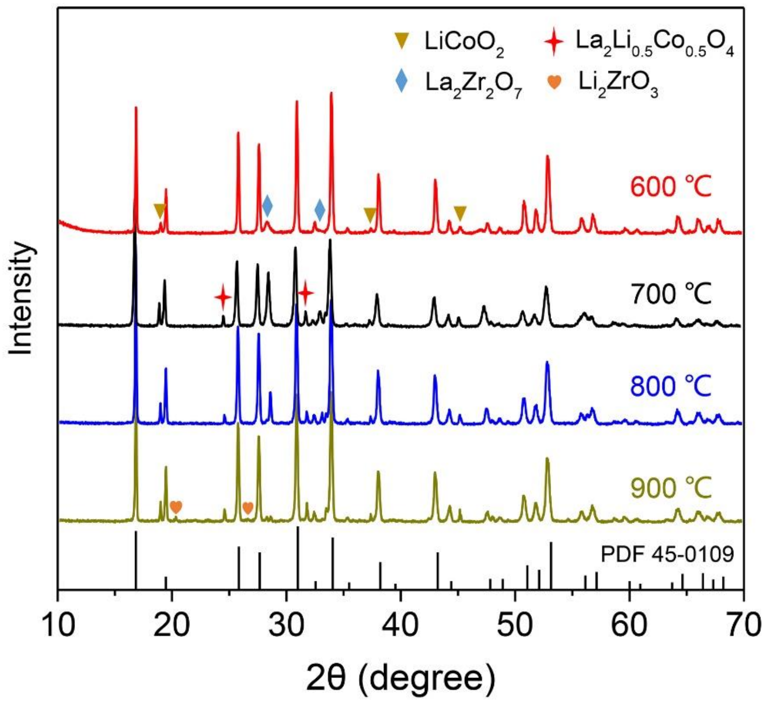

Supplementary Figure 7: XRD patterns of the LLZTO@Li_2CO_3 and Co_3O_4 sintered at different temperatures for 4 hours.

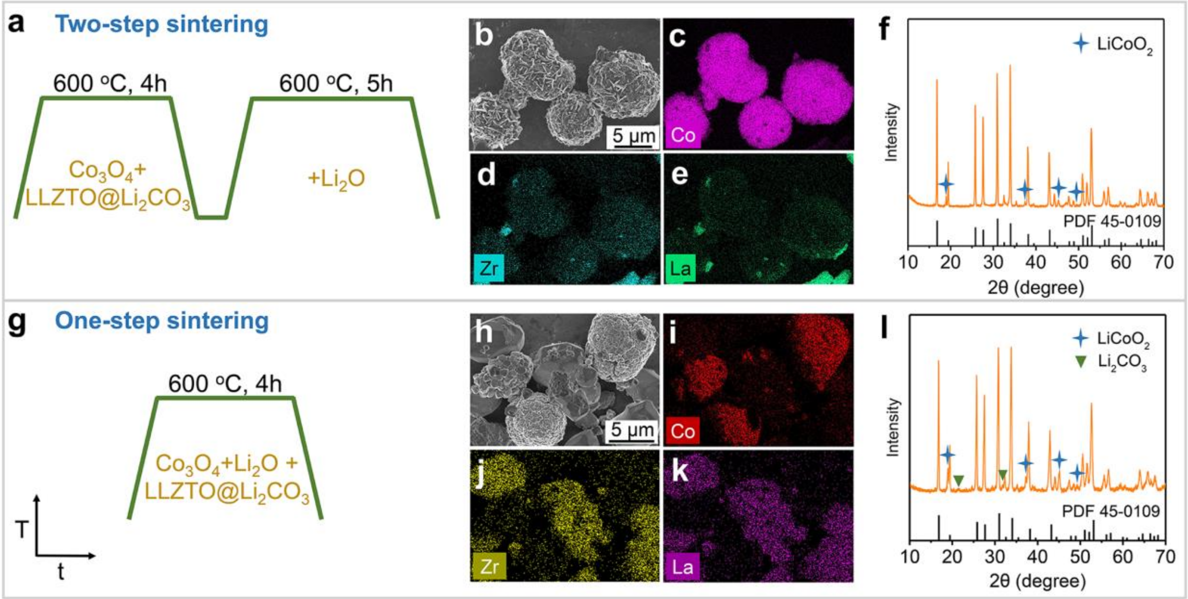

Supplementary Figure 8: Comparison of one-step sintering and two-step sintering. Schematic illustration of **a** two-step sintering and **b** one-step sintering, and corresponding **b,h** SEM images, **c-e, i-k** EDX mapping spectra and **f, l** XRD spectra. The XRD samples were stored in the air for four weeks before testing.

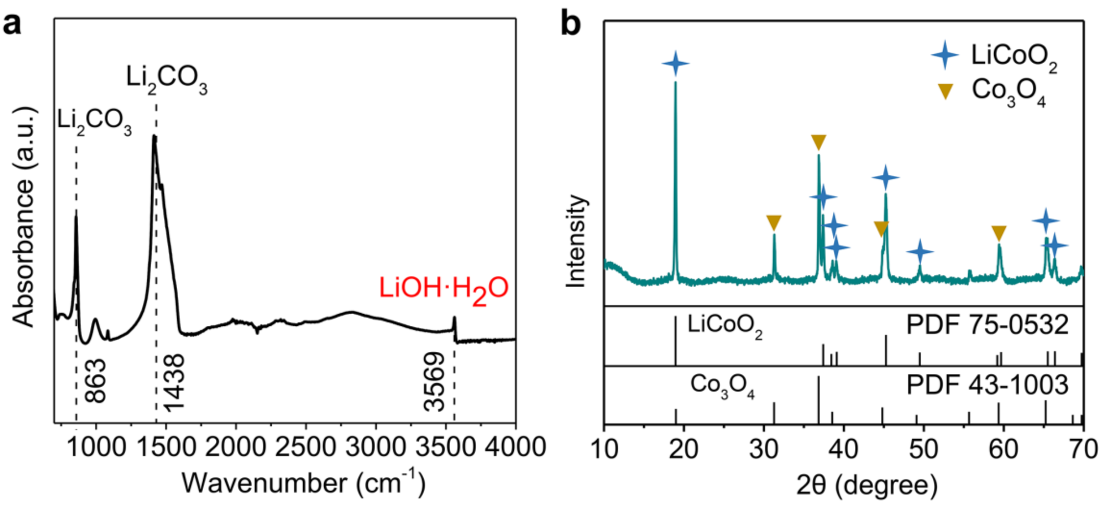

Supplementary Figure 9: Characterization and transformation of LiOH·H_2O. a FTIR spectra of the LLZTO sample exposed to air for four months. There is almost no LiOH·H_2O on the surface of LLZTO, and Li_2CO_3 is dominant. **b** XRD pattern of the LiOH·H_2O and Co_3O_4 sintered at 600 °C for 4 hours. LiOH·H_2O was transformed to $LiCoO_2$ by reacting with Co_3O_4.

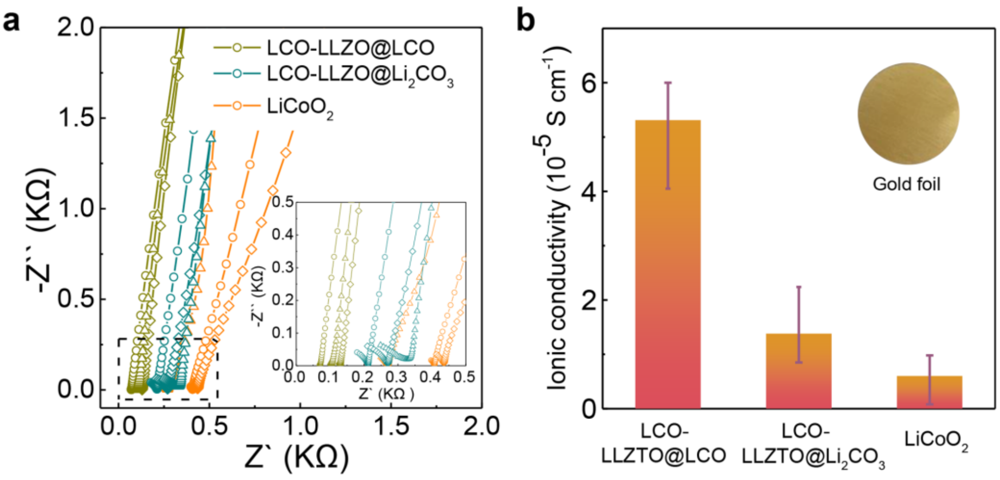

Supplementary Figure 10: EIS analysis of the cathodes without KB. a EIS of Au/Cathode without KB/Au. The cathode slurry without KB was coated on a gold foil, and another gold foil was pressed on the cathode surface after semi-drying, and then dried at 80 ^{o}C for 8h. **b** Conductivity of the cathodes without KB. The conductivity of the cathodes were calculated using $\boldsymbol{\sigma} = \frac{\mathbf{1}}{\mathbf{R}}\frac{\mathbf{L}}{\mathbf{A}}$, where σ is the conductivity of the cathode; R is the total resistance obtained from the Z` value of the inflection point; L and A are the cathode thickness and measurement area, respectively.

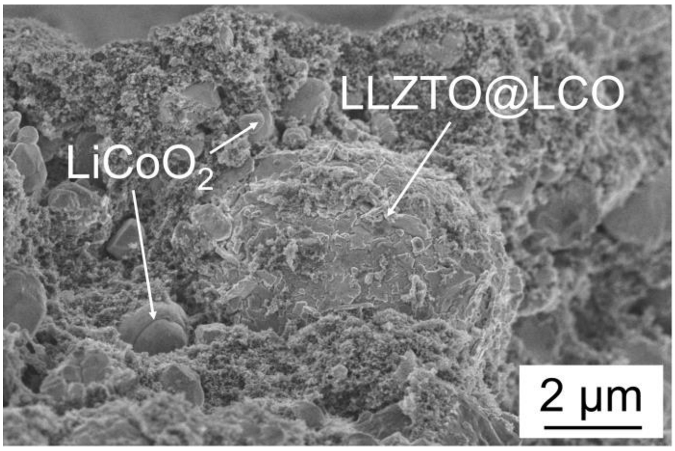

Supplementary Figure 11: SEM image of the LLZTO@LCO particle in the LCO-LLZTO@LCO composite cathode.

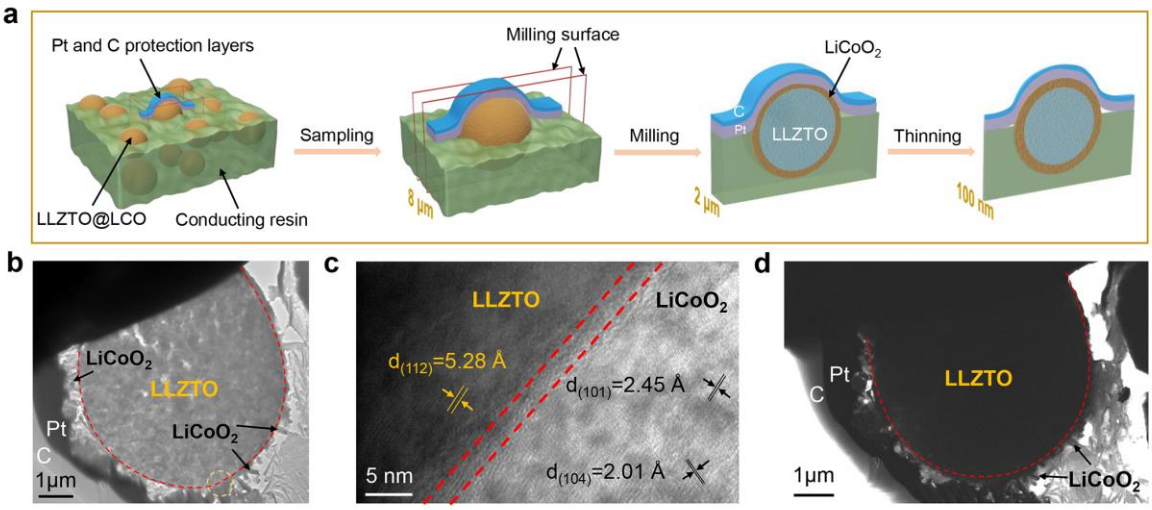

Supplementary Figure 12: Characterization of the interface between LLZTO and $LiCoO_2$ in LLZO@LCO particle. a Schematic illustration of the LLZTO@LCO lamella preparation by using FIB system. **b** TEM, HRTEM and BF-STEM images of the LLZTO@LCO lamella.

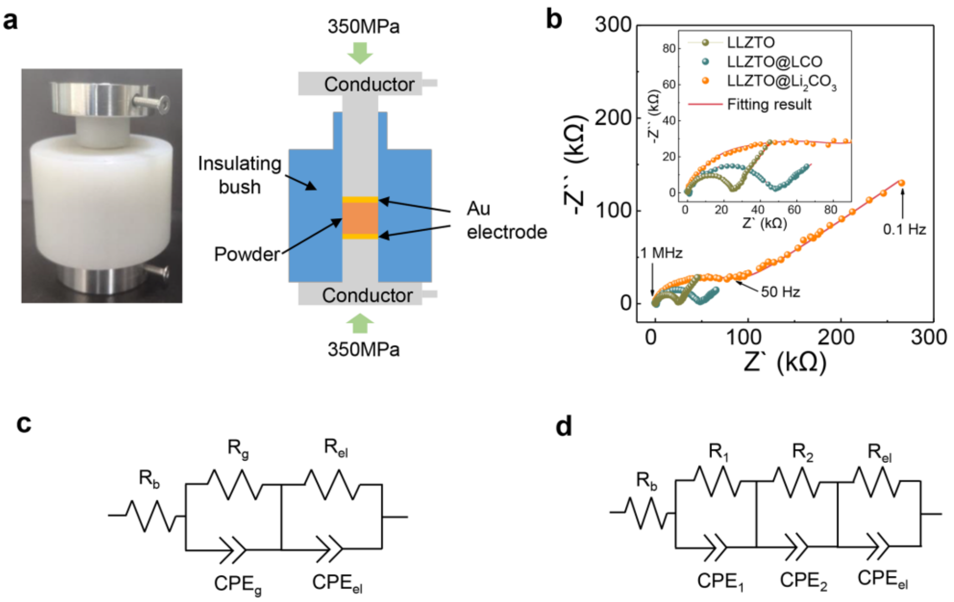

Supplementary Figure 13: EIS analysis of the cold-pressed LLZTO, LLZTO@Li_2CO_3 and LLZTO@LCO pieces. a Physical photo and structure schematic of the cold-pressing mold. **b** EIS of Au/cold-pressed piece/Au samples. **c** Equivalent circuit of Au/LLZTO/Au. **d** Equivalent circuit of Au/LLZTO@LCO/Au and Au/LLZTO@Li_2CO_3/Au.

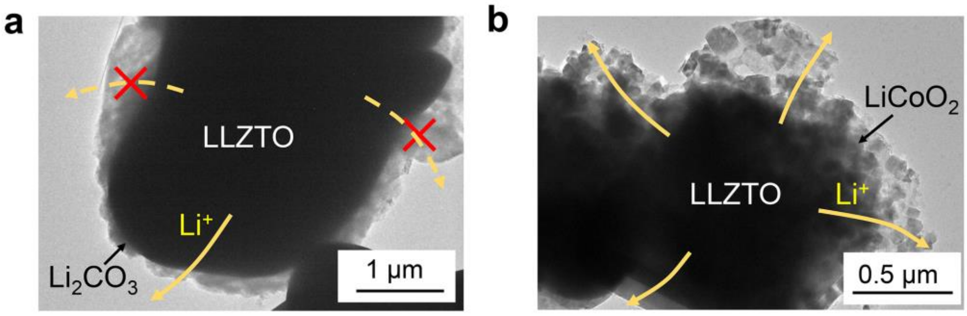

Supplementary Figure 14: Lithium-ion transport paths on the LLZTO@Li_2CO_3 and LLZTO@LCO particles. TEM images of the **a** LLZTO@Li_2CO_3 and **b** LLZTO@LCO particles

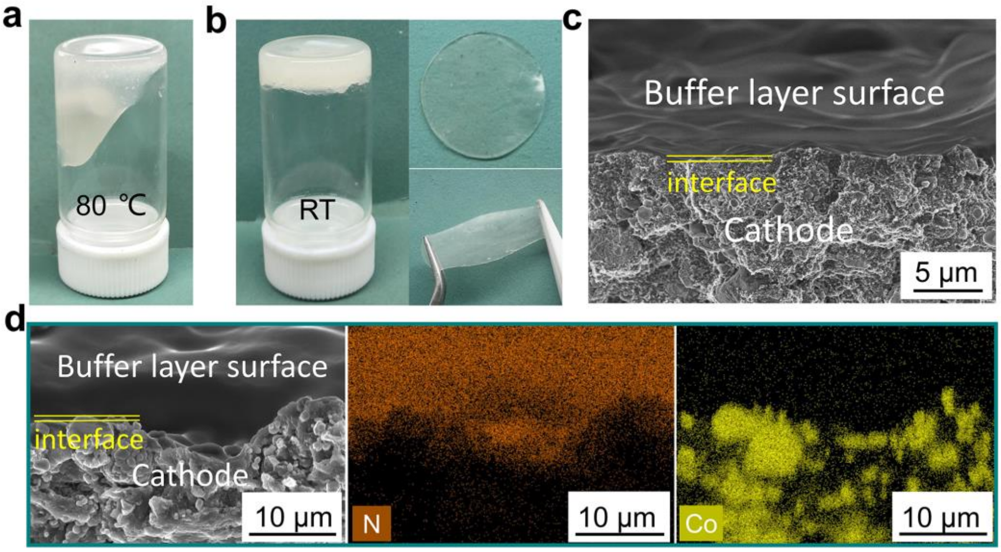

Supplementary Figure 15: Characterization of the buffer layer. a, b Optical photos of the buffer layer at 80 ^{o}C and room temperature. It is semi-fluid with strong viscoelasticity at 80 ^{o}C and is solid with high elasticity at room temperature. **c** SEM image of the composite cathode coated with buffer layer. **d** EDX analysis of the composite cathode coated with buffer layer. The buffer layer is very thin and is clearly layered with the cathode, excluding the possibility that the buffer layer penetrates into the cathode.

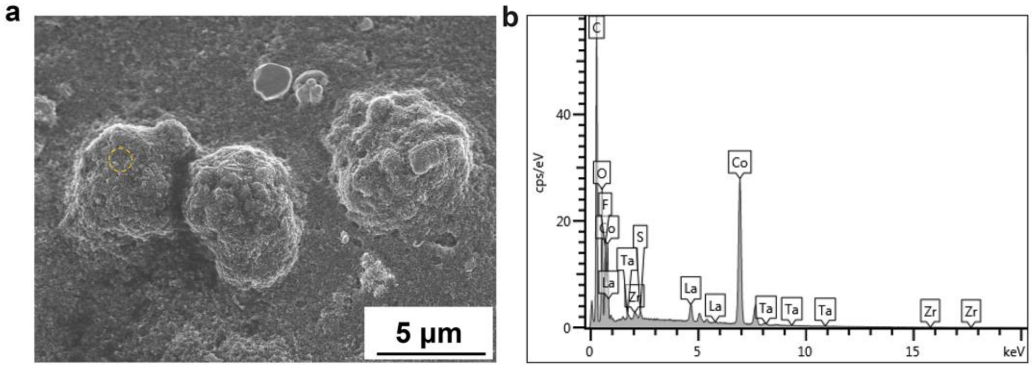

Supplementary Figure 16: Stability of the structure of LLZTO@LCO after charge/discharge 180 cycles. a SEM image of the LLZTO@$LiCO_2$ after 180 cycles in composite cathode. The size of the LLZTO@$LiCoO_2$ particles are the same before and after 180 cycles, to a certain extent, indicating the stability of the structure of LLZTO@$LiCoO_2$, but the surface is covered by the PVDF binder after being added to composite cathode. **b** Element analysis of the region. Zr, La, Ta, Co and O elements come from LLZTO@$LiCoO_2$. C, F elements come from the PVDF binder; A small amount of S elements may come from the LiTFSI in the buffer layer.

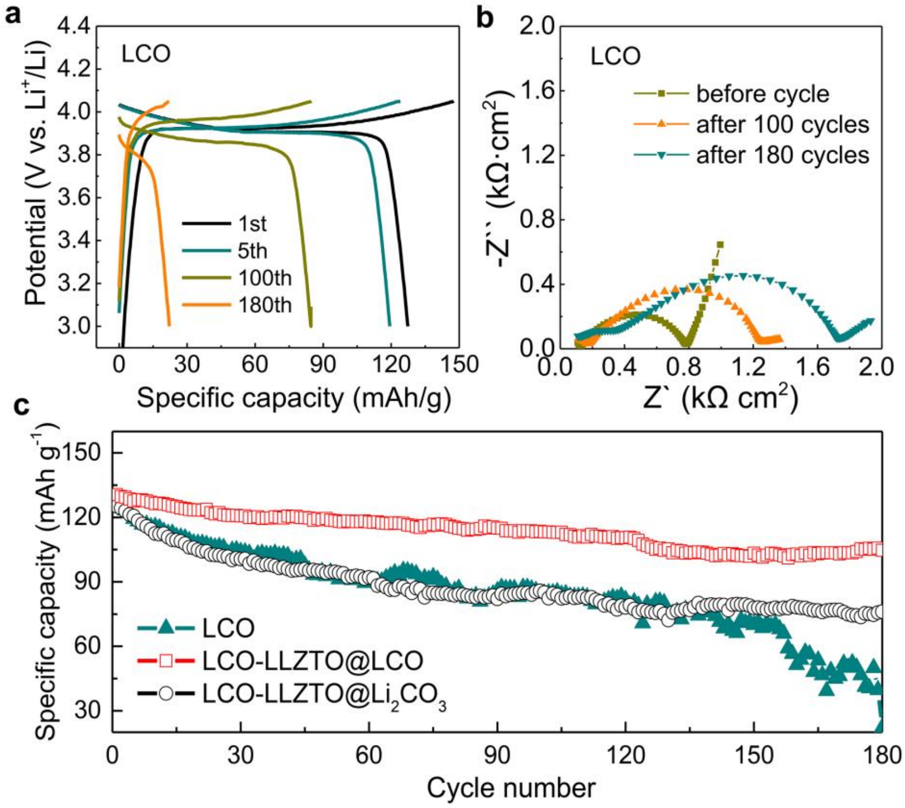

Supplementary Figure 17: Electrochemical performance of the SSB with a commercial $LiCoO_2$ cathode. a, b Discharge/charge curves and EIS of the SSBs with an LCO cathode. **c** Cycling performance of the SSBs with an LCO, LCO-LLZTO@LCO and LCO-LLZTO@Li_2CO_3 cathode, separately. All tests were performed at room temperature.

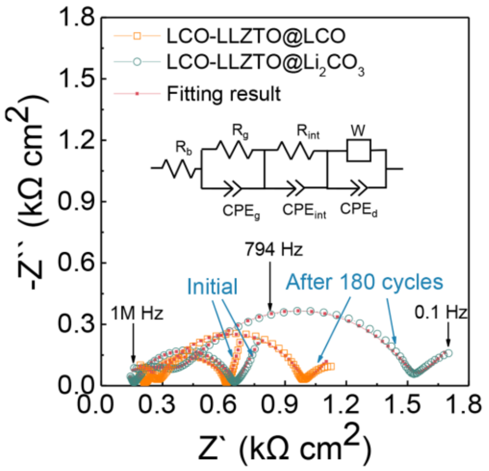

Supplementary Figure 18: Equivalent circuit analysis of EIS for the SSBs with an LCO-LLZTO@LCO and LCO-LLZTO@Li_2CO_3 cathode, separately.

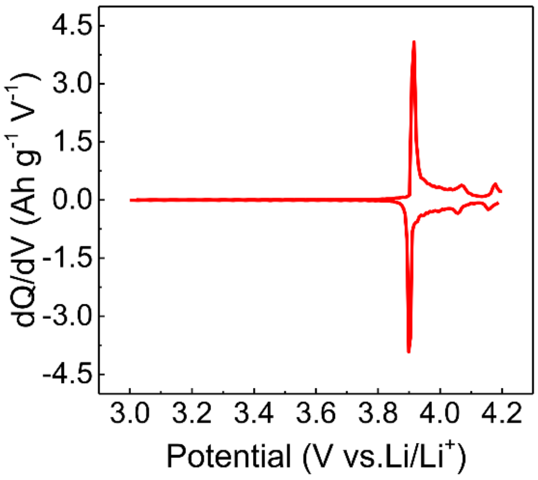

Supplementary Figure 19: dQ dV^{-1} curve of the SSB with an LCO-LLZTO@LCO cathode in the voltage range of 3.0~4.2 V.

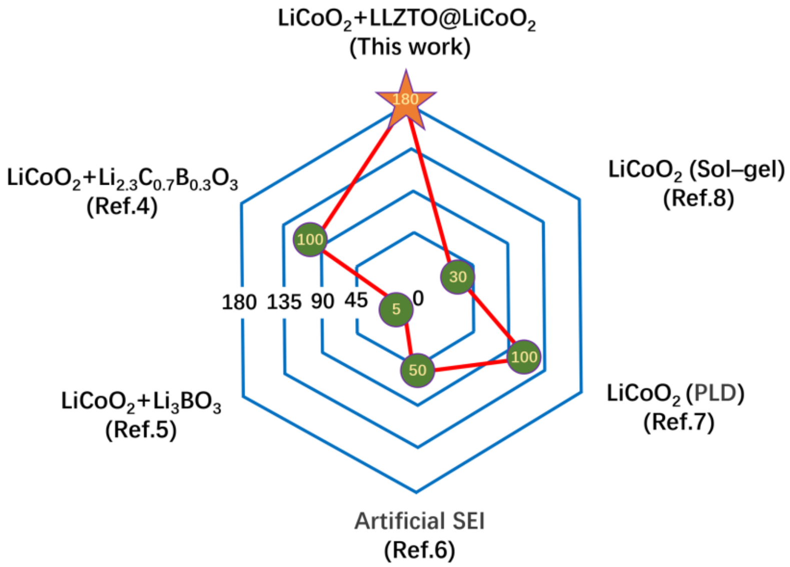

Supplementary Figure 20: Cycle life of various $LiCoO_2$-based SSBs at room temperature.

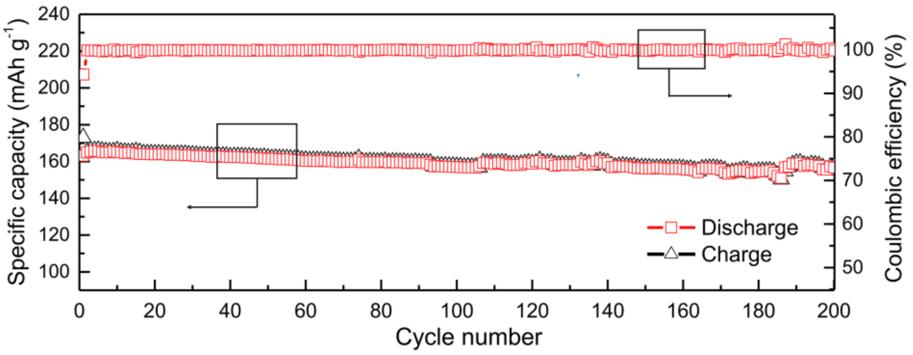

Supplementary Figure 21: Cycling performance of the SSB with an LFP-LLZTO@LCO cathode.

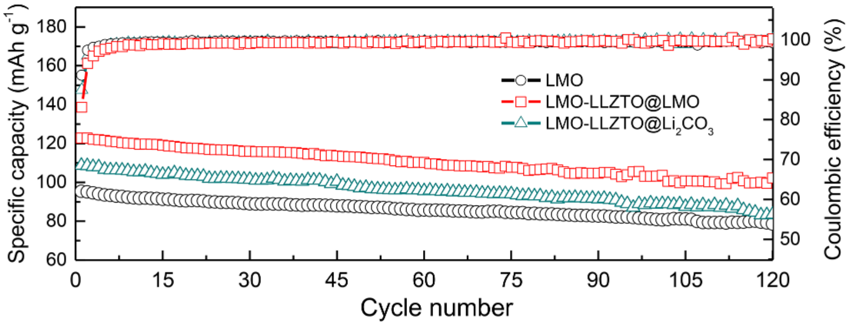

Supplementary Figure 22: Cycling performance of the SSBs with an LMO, LMO-LLZTO@LMO and LMO-LLZTO@Li_2CO_3 cathode.

Supplementary Tables

Supplementary Table 1: Analysis results of equivalent circuits for EIS. R_b is for bulk resistance of LLZTO electrolyte piece. R_g are for grain boundary impedance of LLZTO electrolyte piece. R_{int} and CPE_{int} are for overall interface resistance and double-layer capacitance inside battery. The Warburg impedance (W) and CPE_d are for diffusion impedance.

Composite cathode		R_b Ω cm^2	R_g Ω cm^2	CPE_g × 10^{-9} F cm^2	R_{int} Ω cm^2	CPE_{int} × 10^{-6} F cm^2	CPE_d × 10^{-3} F cm^2
LCO-LLZTO@LCO	Initial	24	166	1.7	360	5.2	7.6
	After 180 cycles	35	202	6.2	600	3.8	9.3
LCO-LLZTO@Li_2CO_3	Initial	23	120	2.7	435	3.5	6.6
	After 180 cycles	30	340	1.7	988	5.0	6.5

Supplementary Table 2: Comparison of the cycle performance of the LLZO-based SSBs employing $LiFePO_4$ cathodes.

Number of cycles	Temperature	Current	Ref.
100	60 °C	0.1 C	9
100	RT	0.1 C	10
50	RT	0.1 C	11
160	65 °C	0.2 C	12
100	60 °C	0.05 C	13
200	90 °C	0.2 C	14
300	40 °C	0.1 mA cm^{-2}	15
200	RT	0.1 C	This work

Supplementary Table 3: Fitting results of Au/cold-pressed piece/Au.

Cold-pressed pieces	R_b (Ω)	R_g (Ω)	CPE_g (10^{-9} F)
LLZTO	19	25057	5.8

Supplementary Table 4: Fitting results of Au/cold-pressed LLZTO@Li_2CO_3/Au.

Cold-pressed piece	R_b (Ω)	R_1 (Ω)	R_2 (Ω)	CPE_1 (10^{-9} F)	CPE_2 (10^{-7} F)
LLZTO@LCO	20	16524	29083	3.3	0.23
LLZTO@Li_2CO_3	27	28500	50252	9.6	5.3

Supplementary Discussions

Supplementary Discussion 1

Supplementary Figure 8a shows the two-step sintering method. From the EDX mapping images (Supplementary Figure 8c-e), the Co, O element are uniformly distributed on the surface of particles and the La, Zr elements can also be detected in that region, corresponding to the LLZTO@$LiCoO_2$ structure. Moreover, the SEM image shows that $LiCoO_2$ on the surface of LLZTO exhibits a nanoplate-like (Supplementary Figure 8b), which is beneficial to the conduction of lithium ions inside cathode. The XRD pattern shows the composition of sintered materials stored in the air for four weeks. There are no peaks of Li_2CO_3, showing that it was not regenerated due to the protection of $LiCoO_2$ shell (Supplementary Figure 8f). Supplementary Figure 8g shows the one-step sintering method. The EDX mapping images show that the Co element is distributed in a region separately, where Zr or La elements are not observed (Supplementary Figure 8i-k), indicate that the LLZTO@$LiCoO_2$ coating structure was not formed. This is because in the one-step sintering method, Co_3O_4 is in contacts with a large amount of Li_2O directly and tends to react preferentially to form separate $LiCoO_2$ particles. Conversely, in the two-step sintering method, the contact between Co_3O_4 and Li_2O is eliminated, and Co_3O_4 can only react with Li_2CO_3 to form a $LiCoO_2$ coating on the surface of LLZTO. As shown in SEM image (Supplementary Figure 8h), the irregular particles with a smooth surface are pure LLZTO, and the spherical blocks stacked with small particles are pure $LiCoO_2$, which agree with the results of EDX. Correspondingly, Li_2CO_3 still exists in store due to the direct contact of the LLZTO surface with air as shown in XRD pattern (Supplementary Figure 8l).

Supplementary Discussion 2

Supplementary Figure 12a shows the preparation process of LLZTO@LCO lamella sample. Firstly, platinum (Pt) and carbon (C) protection layers are deposited onto the surface of an LLZTO@LCO particle by FIB system before lamella preparation to avoid damage from the Ga-ion milling. Then, the block is milled around the LLZTO@LCO particle by FIB to take out the preliminary sample (cuboid area drawn by red line). Subsequently, the sample is gradually milled along the milling surfaces on both sides of sample to thin it. After the milling surfaces are polished, the LLZTO@LCO lamella with a thickness of about 100 nm is prepared. Supplementary Figure 12b shows the TEM image of the LLZTO@LCO lamella. It can be seen that there are many $LiCoO_2$ nano-fragments distributed along the surface of LLZTO. Meanwhile, this distribution is also clearly observed in the bright-field (BF) scanning transmission electron microscopy (STEM) image (Supplementary Figure 12d). But, it also can be seen that a small part of the LLZTO surface is not covered by $LiCoO_2$, This may be caused by the damage of Ga-ion beam to the surface layer during lamella preparation. Additionally, from the HRTEM image (Supplementary Figure 12c), we can observe that there is a relatively chaotic atomic arrangement at the interface between LLZTO and $LiCoO_2$ within a range of about 3 nm, implying the atoms are interlaced at the junction of LLZTO and $LiCoO_2$, which leads to a tightly connected interface and facilitates the transfer of lithium ions between LLZTO and $LiCoO_2$, thereby increasing the diffusion rate of lithium ions inside cathode. The lattice spacing of 0.528 nm agrees with the (112) facets of LLZTO, and the lattice spacing of 0.245 and 0.201 nm agrees with the (101) and (104) facets of $LiCoO_2$ respectively.

Supplementary Discussion 3

Electrochemical tests of the cold-pressed LLZTO, LLZTO@Li_2CO_3 and LLZTO@LCO pieces were undertaken to analyze the ion-transport properties at the LLZTO/LCO interface. This method minimizes the type of interface inside test samples by excluding the use of other additives, lead to the LLZTO/LCO interface can be distinguished in EIS. As shown in Supplementary Figure 13a, LLZTO, LLZTO@Li_2CO_3 and LLZTO@LCO powders were filled into the insulating bush and pressed into pieces at room temperature, respectively. Gold foils were used as the blocking electrodes. The EIS tests of the cold-pressed pieces were performed under 350 MPa and 80 ^oC. Supplementary Figure 13b shows the normalized (thickness is taken into account) EISs of LLZTO, LLZTO@Li_2CO_3 and LLZTO@LCO cold-pressed pieces, respectively. The impedance plot of Au/LLZTO/Au contains an obvious semicircle at high frequency region and a slanted tail at low frequency region. The corresponding equivalent circuit is shown in Supplementary Figure 13c. R_b and R_g are for bulk and grain boundary resistance of cold-pressed electrolyte piece, respectively. CPE_g is for double-layer capacitance at grain boundary. R_{el} and CPE_{el} are for the resistance of Au electrode and the double-layer capacitance at the interface between LLZTO and Au electrode. The fitting results are shown in the Supplementary Table 3. It can be seen that LLZTO cold-pressed piece shows a grain boundary resistance of R_g (25057 Ω), which can be attributed to the grain boundary of LLZTO/LLZTO. However, the impedance plots of Au/LLZTO@LCO/Au and Au/LLZTO@Li_2CO_3/Au show a flat semicircle at high frequency region, which is due to the presence of the LCO and Li_2CO_3 surface layer resulting in the addition of a poorly-resolved semicircle. Their equivalent circuit is shown in Supplementary Figure 13d. R_1 and CPE_1 are for LLZTO/LCO interface in LLZTO@LCO particle or LLZTO/Li_2CO_3 interface in LLZTO@Li_2CO_3 particle. R_2 and CPE_2 are for LCO/LCO interface between LLZTO@LCO particles or Li_2CO_3/Li_2CO_3 interface between LLZTO@Li_2CO_3 particles.The fitting results are shown in the Supplementary Table 4. It can be seen that the LLZTO@Li_2CO_3 cold-pressed piece shows an interfacial resistance of R_1 (28500 Ω) and an interfacial resistance of R_2 (50252 Ω), corresponding to LLZTO/Li_2CO_3 and Li_2CO_3/Li_2CO_3 interfaces respectively, indicating slow ion-transport between LLZTO/Li_2CO_3 particles. In contrast, the LLZTO@LCO cold-pressed piece shows a smaller LLZTO/LCO interface resistance R_1=15524 Ω and LCO/LCO interface resistance R_2=29083 Ω, confirming that the converted LLZTO/LCO interface enables faster lithium-ion transfer.

Supplementary References

1 Wang, C., & Hong, J. Ionic/electronic conducting characteristics of $LiFePO_4$ cathode materials the determining factors for high rate performance. *Electrochem. Solid State Lett.* **10**, 65-69 (2007).

2 Thangadurai, V., Huggins, R., & Weppner, W. Use of simple ac technique to determine the ionic and electronic conductivities in pure and Fe-substituted $SrSnO_3$ perovskites. *J. Power Sources* **108**, 64-69 (2002).

3 Huggins, R. Simple method to determine electronic and ionic components of the conductivity in mixed conductors a review. *Ionics* **8**, 300-313 (2002).

4 Han, F. *et al.* Interphase engineering enabled all-ceramic lithium battery. *Joule* **2**, 497-508 (2018).

5 Ohta, S. *et al.* All-solid-state lithium ion battery using garnet-type oxide and Li_3BO_3 solid electrolytes fabricated by screen-printing. *J. Power Sources* **238**, 53-56 (2013).

6 Fan X *et al.* Fluorinated solid electrolyte interphase enables highly reversible solid-state Li metal battery. *Sci. Adv.* **4**, eaau9245 (2018).

7 Ohta, S., Kobayashi, T., Seki, J. & Asaoka, T. Electrochemical performance of an all-solid-state lithium ion battery with garnet-type oxide electrolyte. *J. Power Sources* **202**, 332-335 (2012).

8 Kotobuki, M. & Kanamura, K. Fabrication of all-solid-state battery using $Li_5La_3Ta_2O_{12}$ ceramic electrolyte. *Ceram. Int.* **39**, 6481-6487 (2013).

9 Yan, X., Li, Z., Wen, Z., & Han, W. Li/$Li_7La_3Zr_2O_{12}$/$LiFePO_4$ all-solid-state battery with ultrathin nanoscale solid electrolyte. *J. Phys. Chem. C* **121**, 1431-1435 (2017).

10 He, M., Cui, Z., Chen, C., Li, Y. & Guo, X. Formation of self-limited, stable and conductive interfaces between garnet electrolytes and lithium anodes for reversible lithium cycling in solid-state batteries. J. Mater. Chem. A **6**, 11463-11470 (2018).

11 Lu, Z. *et al.* Enabling room-temperature solid-state lithium-metal batteries with fluoroethylene carbonate-modified plastic crystal interlayers. *Energy Storage Mater.* **18**, 311-319 (2019).

12 Zhou, W. *et al.* Polymer lithium-garnet interphase for an all-solid-state rechargeable battery. *Nano energy* **53**, 926-931 (2018).

13 Du, F. et al. All solid state lithium batteries based on lamellar garnet-type ceramic electrolytes. J. Power Sources **300**, 24-28 (2015)

14 Chia, S., Liu, Y., Zhao, N., Guo, X., Nan, C. & Fan, L. Solid polymer electrolyte soft interface layer with 3D lithium anode for all-solid-state lithium batteries. *Energy Storage Mater*. **17**, 309-316 (2019).

15 Xu, H. *et al*. Li_3N-modified garnet electrolyte for all-solid-state lithium metal batteries operated at 40 C. *Nano Letters* **18**, 7414-7418 (2018).
